# Application and Comparison of Machine Learning and Database-Based Methods in Taxonomic Classification of High-Throughput Sequencing Data

**DOI:** 10.1093/gbe/evae102

**Published:** 2024-05-15

**Authors:** Qinzhong Tian, Pinglu Zhang, Yixiao Zhai, Yansu Wang, Quan Zou

**Affiliations:** Institute of Fundamental and Frontier Sciences, University of Electronic Science and Technology of China, Chengdu, China; Yangtze Delta Region Institute (Quzhou), University of Electronic Science and Technology of China, Quzhou 324003 China; Institute of Fundamental and Frontier Sciences, University of Electronic Science and Technology of China, Chengdu, China; Yangtze Delta Region Institute (Quzhou), University of Electronic Science and Technology of China, Quzhou 324003 China; Institute of Fundamental and Frontier Sciences, University of Electronic Science and Technology of China, Chengdu, China; Yangtze Delta Region Institute (Quzhou), University of Electronic Science and Technology of China, Quzhou 324003 China; Institute of Fundamental and Frontier Sciences, University of Electronic Science and Technology of China, Chengdu, China; Yangtze Delta Region Institute (Quzhou), University of Electronic Science and Technology of China, Quzhou 324003 China; Institute of Fundamental and Frontier Sciences, University of Electronic Science and Technology of China, Chengdu, China; Yangtze Delta Region Institute (Quzhou), University of Electronic Science and Technology of China, Quzhou 324003 China

**Keywords:** taxonomic classification, machine learning, metagenomics, comparison, database

## Abstract

The advent of high-throughput sequencing technologies has not only revolutionized the field of bioinformatics but has also heightened the demand for efficient taxonomic classification. Despite technological advancements, efficiently processing and analyzing the deluge of sequencing data for precise taxonomic classification remains a formidable challenge. Existing classification approaches primarily fall into two categories, database-based methods and machine learning methods, each presenting its own set of challenges and advantages. On this basis, the aim of our study was to conduct a comparative analysis between these two methods while also investigating the merits of integrating multiple database-based methods. Through an in-depth comparative study, we evaluated the performance of both methodological categories in taxonomic classification by utilizing simulated data sets. Our analysis revealed that database-based methods excel in classification accuracy when backed by a rich and comprehensive reference database. Conversely, while machine learning methods show superior performance in scenarios where reference sequences are sparse or lacking, they generally show inferior performance compared with database methods under most conditions. Moreover, our study confirms that integrating multiple database-based methods does, in fact, enhance classification accuracy. These findings shed new light on the taxonomic classification of high-throughput sequencing data and bear substantial implications for the future development of computational biology. For those interested in further exploring our methods, the source code of this study is publicly available on https://github.com/LoadStar822/Genome-Classifier-Performance-Evaluator. Additionally, a dedicated webpage showcasing our collected database, data sets, and various classification software can be found at http://lab.malab.cn/~tqz/project/taxonomic/.

**Significance**

In the burgeoning field of bioinformatics, efficiently classifying species from vast amounts of sequencing data remains a central challenge. Our research conducted an exhaustive comparison between database-based and machine learning methods for this task, unveiling their individual strengths and weaknesses. By integrating these approaches, we provide a novel strategy that enhances taxonomic classification, promising significant advances in biodiversity research and other related biological disciplines.

## Introduction

As high-throughput sequencing technology continues to evolve, taxonomic classification has emerged as a cornerstone in the field of bioinformatics (Hassemer et al. 2019). Accurate taxonomic classification is not merely an academic concern; it is crucial for uncovering biological diversity, conducting disease-associated metagenome studies, and undertaking environmental surveillance (Uyaguari-Diaz et al. 2016; Kim et al. 2017; Nooij et al. 2018). Yet, efficiently processing and analyzing the sheer volume of sequencing data for precise taxonomic classification remains a daunting task (Gardiner et al. 2021).

In the landscape of existing taxonomic classification techniques, the dominant paradigms are database-based (DB) and machine learning (ML) methods. DB approaches primarily focus on aligning sequencing reads against a known reference genome database to determine taxonomic classification (Bonin et al. 2023). These methods require substantial hard drive space to store the database and a significant amount of memory to load the database for use (Wright et al. 2023). In contrast, ML techniques endeavor to classify species by discerning patterns within the training data set and thereby making predictions (Liang et al. 2020). Typically, these methods only need a much smaller model, making them more efficient in terms of storage and memory usage.

Each of these methodologies comes with its own set of merits and drawbacks. While DB methods can achieve higher classification precision when the reference database is extensive and complete, they are constrained by the quality and scope of the reference database itself (Parks et al. 2020). ML approaches, on the other hand, offer advantages when reference sequences are sparse or lacking, as they can extrapolate the existence of unknown species from the training data; however, their performance is often limited by the representativeness and volume of the training data (Mock et al. 2022).

In light of these complexities, our study sets out to not only compare DB methods with ML approaches but also to investigate the feasibility of integrating multiple DB techniques. This multifaceted research aims to rigorously assess the performance and suitability of these methods using simulated data sets. Ultimately, our objective is to explore avenues for optimizing and amalgamating these techniques, with the goal of elevating the accuracy and efficiency of future taxonomic classification efforts.

### DB Taxonomic Classification Method

DB taxonomic classification methods hold a pivotal position in bioinformatics and constitute a major trend in contemporary classification strategies (Ames et al. 2013). Utilizing reference databases, these methods determine the taxonomic affiliations of unknown sequences; their accuracy and efficiency are strongly influenced by both the comparison strategies employed and the quality of the databases themselves (Portik et al. 2022). With the surge in high-throughput sequencing data, the task of processing and analyzing large data sets for accurate classification has become increasingly challenging (Johnson et al. 2019; Yang et al. 2020). To tackle this, DB methods deploy sophisticated algorithms and computations, serving as a potent solution. These methods are broadly categorized into three primary types: methods based on Alignment, methods based on Markers, and methods based on k-mer. Each category exhibits its own unique features, possesses applicability across diverse scenarios, and thus manifests the adaptability and richness of the field.

#### Alignment-Based Methods

Alignment-based taxonomic classification methods are pivotal in bioinformatics, facilitating species identification by aligning unknown sequences against known sequences in reference databases. These methods are applied extensively across various domains, including evolutionary biology and metagenomics. With technological advancements, sophisticated sequence alignment techniques have been developed to enhance efficiency and accuracy (Gao et al. 2017; Furstenau et al. 2022). These advanced methodologies offer more precise measurements of sequence similarity, thereby improving classification accuracy. However, the computational complexity of these methods remains relatively high, particularly for large-scale data sets, necessitating significant computational resources. Alignment-based methods have wide applications in bioinformatics, and ongoing technological improvements are expected to further enhance their efficiency and applicability for research purposes (Eisenhofer and Weyrich 2019; Zhang et al. 2024).

#### Marker-Based Methods

In microbiomics and bioinformatics, marker-based classification methodologies leverage highly conserved marker genes or proteins within specific species or groups for species identification (Tovo et al. 2020; Hugenholtz et al. 2021). These approaches achieve precise species determination by comparing marker gene sequences from unknown samples with known sequences in reference databases, exemplified by the widespread use of the 16S rRNA gene in bacterial classification (Lan et al. 2016; Vicente Dos Santos and Tixier 2017). While these marker-based classification methods have gained recognition in the scientific community, the increasing volume and diversity of sequencing data necessitate further research and innovation to address challenges in identifying unknown or rare microbes and managing the surge in data storage and retrieval demands (Blanco-Míguez et al. 2023).

#### k-mer-Based Method

In bioinformatics, the taxonomic classification method based on k-mer is a vital classification technique, which complements the methods based on Alignment and Marker. In this approach, k-mer refers to a DNA sequence fragment with a length of k. Such fragments serve as feature units in species’ genomic data. By analyzing the occurrence frequency of k-mer in diverse species’ genomes, taxonomic classification is achieved (Shaw and Yu 2022). The merits of this method are its efficiency, universality, and expandability. As the count of k-mers is finite, structures like hash tables can be utilized for storing and searching k-mers, enabling swift taxonomic classification (Breitwieser et al. 2018). Additionally, the k-mer approach is not dependent on particular genes or genomic structures, allowing its application to any species’ genomic data. Kraken is a notable application example of the k-mer-based classification method (Wood and Salzberg 2014). At the core of Kraken is a database containing k-mers and their lowest common ancestor (LCA). It quickly assigns a classification to each k-mer by matching it with the most specific node in the taxonomic tree associated with the given k-mer. This efficient approach is also utilized by some other traditional k-mer-based classification software, which similarly employs the LCA for taxonomy resolution (Menzel et al. 2016; Piro et al. 2020).

Nonetheless, the k-mer method has its restrictions. Initially, this technique presumes that every genome region equally contributes to taxonomic classification. Yet, diverse gene regions might have varied contributions (Han and Cho 2019). Next, the k-mer approach is exceptionally sensitive to sequence inaccuracies; slight errors can result in numerous k-mer mistakes (Vinje et al. 2015). Ultimately, the k-mer method can not address intricate biological events like gene recombination or horizontal gene transfers (Dubinkina et al. 2016).

**Fig. 1.**
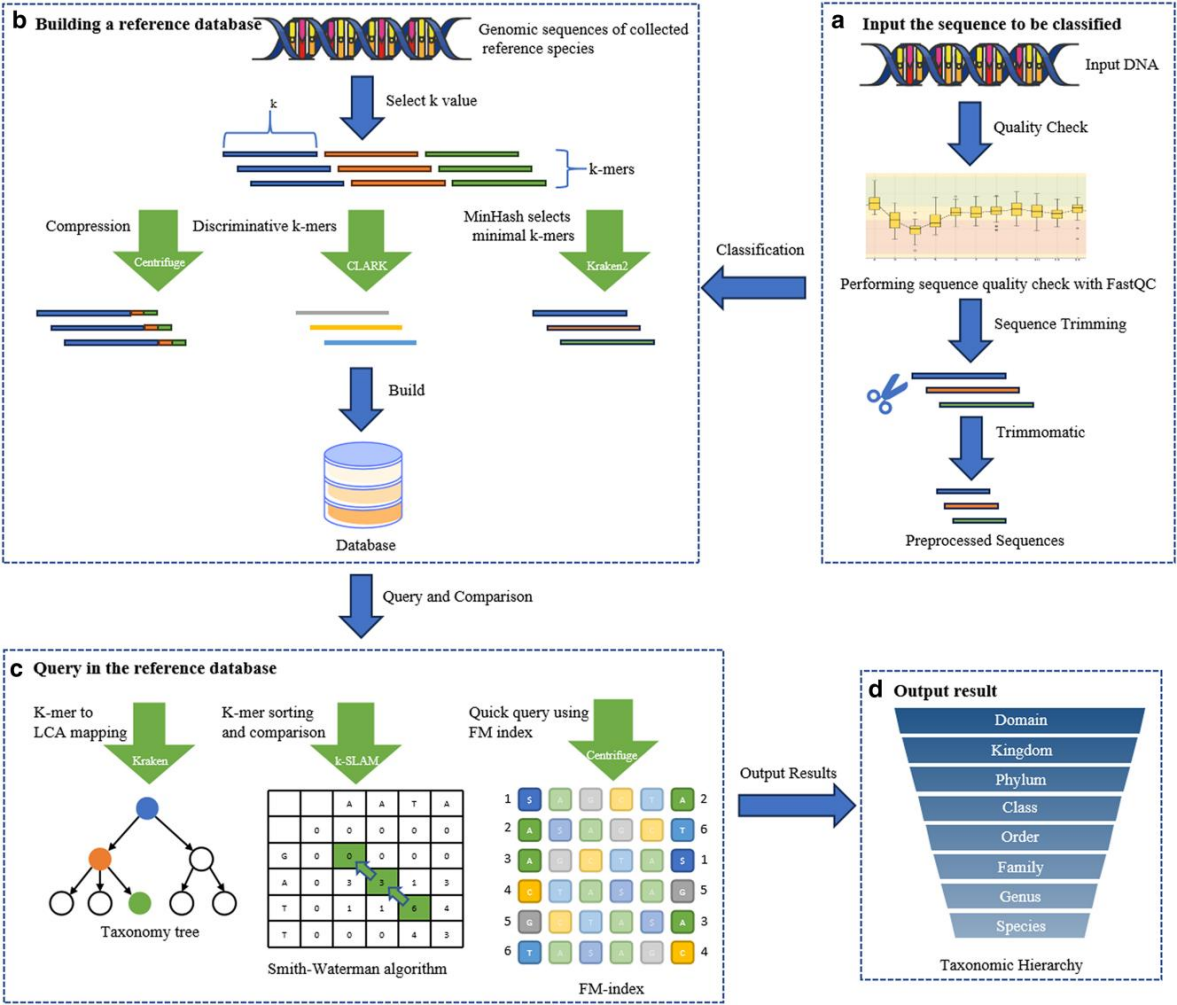
Flowchart of taxonomic classification based on k-mer and software enhancement strategies. The arrows with smaller heads illustrate the conventional k-mer classification pathway, encompassing the formulation of databases and the incorporation of DNA sequences leading to the classification outcome. The wider arrows highlight the unique optimization techniques implemented by specific software, along with the software names. a) The preprocessing of the genetic sequences to be classified, including Quality Check and Sequence Trimming. b) The construction of reference databases by species classification software, detailing unique optimization methods employed by three different software tools during database construction. c) The querying of input sequences within the reference databases, listing the querying methods of three software tools. d) The output of classification results, which are hierarchical classifications ranging from domain to species.

Overall, the k-mer-based taxonomic classification technique is potent and adaptable. In combination with the Alignment- and Marker-based methods, it offers a holistic and precise taxonomic classification solution. Yet, this method presents its challenges and limitations. It demands additional research and enhancements to cater to varying application contexts and requirements. For a comprehensive understanding of this procedure and potential optimization tactics, we present a flowchart (Fig. 1) illustrating the conventional k-mer classification process from database establishment to DNA sequence input and results, along with unique optimization techniques used by certain software.

To address the limitations of the k-mer taxonomic classification method, a variety of improvement strategies have been proposed by researchers. These are aimed at enhancing both its accuracy and efficiency. Significantly, these improvement strategies are tailored not just for the unique features of the k-mer method itself but also consider the synergistic effects when used in conjunction with methods based on Alignment and Marker genes. Below, we outline some primary directions for improvement.

##### Opting for Specific Data Structures Serves as One Effective Strategy

For example, Kraken2 has managed to increase its query speed while simultaneously reducing memory consumption by adopting compact hash tables and utilizing storage structures like minimizers (Lu and Salzberg 2020). Similarly, KrakenUniq employs the HyperLogLog data structure to estimate the unique number of k-mers in each classification unit, yielding significant benefits in memory efficiency, calculation speed, and accuracy (Breitwieser et al. 2018). Centrifuge leverages the FM index data structure, based on the Burrows–Wheeler transform (BWT), enabling quick searches for k-mers of any length and thus facilitating efficient sequence categorization (Kim et al. 2016). In the case of MegaBLAST, its core feature lies in the strategic organization of database indices, coupled with the deployment of the “seed search strategy.” This allows for the identification of initial matching substrings within both queries and databases (Morgulis et al. 2008). This arrangement encompasses compressed sequence information and positional data for k-mers, while also integrating unique offset and prefix management strategies. Such a setup enables MegaBLAST to efficiently process k-mers, ensuring that the identified seeds adhere to the seed qualification criteria. This innovative data structure furnishes MetaOthello with notable advantages in terms of both memory and computation, facilitating quick and accurate metagenome read classifications (Liu et al. 2018).

##### Optimize k-mer Selection

For instance, CLARK's hallmark feature is its employment of target-specific or distinguishing k-mers for categorization (Ounit et al. 2015). By constructing the target sequence's k-spectrum and eliminating shared k-mers, CLARK opts for k-mers that uniquely and unambiguously represent each target for classification. This focused use of selective k-mers significantly enhances the precision and speed of classification. Similarly, taxMaps’ core strategy aims to optimize k-mer selection and management through a unique compression algorithm, thereby achieving efficient species categorization (Corvelo et al. 2018). This compression strategy not only eradicates database redundancy but also streamlines the categorization process by carrying out LCA pre-allocation and k-mer collapsing, thereby facilitating approximate searches and improving query efficiency. Kaiju introduces a complementary method to this k-mer optimization by focusing on protein-level sequence comparison for classification (Menzel et al. 2016). Utilizing the BWT for rapid and precise string matching, Kaiju surpasses traditional k-mer-based approaches by identifying maximum exact matches (MEMs) at the protein level, thereby enhancing recall especially for underrepresented genera in the database. This capability allows Kaiju to classify more reads with higher accuracy. Additionally, Kaiju's greedy heuristic search mode, which permits a certain number of amino acid substitutions at the match termini, further boosts recall, making it a robust tool for sequence classification at the protein level.

##### Incorporating Intricate Algorithms

For example, Kraken achieves high precision and rapid classification rates by synergistically merging the k-mer approach with the LCA strategy (Wood and Salzberg 2014). k-SLAM employs k-mers for initial alignment but goes a step further by additionally integrating Smith–Waterman pairwise alignment and pseudo-assembly techniques to further boost accuracy (Ainsworth et al. 2017). These additional computational layers make k-SLAM's algorithm more intricate compared with conventional k-mer-based methods. MMseqs2, on the other hand, takes a more comprehensive approach. It does not solely rely on k-mers for matching; instead, it employs a complex strategy that involves first identifying target sequences with two sequential analogous k-mer matches on a single diagonal and then leveraging the BWT for exact string alignments (Steinegger and Söding 2017). These multi-step tactics make MMseqs2's approach both more intricate and more efficient compared with traditional k-mer-centric methods. Additionally, MMseqs2 facilitates iterative profile-to-sequence and sequence-to-profile queries, adding yet another layer to its algorithmic complexity. PathSeq further contributes to this field by employing a unique classification method that begins with the removal of low-quality, low-complexity host-derived reads (Walker et al. 2018). It utilizes a Bloom filter for rapid k-mer searches to detect short sequences in the host reference sequences, effectively eliminating a significant portion of host reads before sequence alignment. The remaining nonhost reads are then mapped to a microbial genome reference library using the BWA–MEM aligner, with paired reads required to map to the same microorganism to enhance specificity. These steps, including parallel data processing, improved low-complexity sequence filtering algorithms, and efficient alignment tools, greatly accelerate analysis speed and efficiency, setting PathSeq apart from traditional methods.

##### Unique k-mer Approach

For example, CLARK-S and DIAMOND, both leveraging spaced seeds, exemplify this innovation. CLARK-S employs a method called spaced k-mers, which allows for mismatches at predefined positions, thereby enhancing the tool's recall without compromising specificity (Ounit and Lonardi 2016). This approach facilitates more flexible and accurate sequence matching, especially useful in complex metagenomic data sets. Similarly, DIAMOND introduces a dual indexing and spaced seed strategy for protein sequence alignment, significantly improving upon traditional continuous seed methods. By identifying all seeds and their positions in both query and reference sequences and then linearly traversing the lists to find all matching seeds, DIAMOND enhances data locality and reduces memory bandwidth requirements. Its use of longer seeds, with only a subset of positions being utilized, strikes a balance between speed and recall, enabling faster alignments without sacrificing accuracy (Buchfink et al. 2015).

These improved strategies showcase the diversity and flexibility of k-mer taxonomic classification methods. By strategically combining diverse technologies and strategies, the precision and performance of k-mer methods can be advanced to new heights. Nevertheless, these enhancements come with new challenges and concerns that demand continued investigation and rigorous probing.

In the face of intricate and varied genomic data, k-mer taxonomic classification techniques have evolved into an essential instrument for researchers. Their high efficiency and generality have led to their extensive application in diverse settings and fields, ranging from environmental genomics to metagenomics, epidemiology, and origin tracking, to name a few (Van Etten et al. 2023). More importantly, k-mer methods do not rely on specific genes or genomic structures, which is particularly beneficial for genomes without detailed annotations or those that are highly heterogeneous.

### ML-Based Taxonomic Classification Methods

In some cases, traditional classification methods can be inefficient and may struggle to recognize new species, particularly when tasked with processing massive amounts of data (Borba et al. 2021). Transitioning from this challenge, ML has recently emerged as a potent computational tool across various scientific and engineering domains, notably including taxonomic classification. ML introduces a novel method for identifying and categorizing unknown biological entities, particularly in the fields of viral genomics and microbiomics (Bartlett et al. 2022). Employing a range of sophisticated algorithms and statistical methodologies, ML can adeptly distill features from intricate biological data, thereby leading to precise categorizations (Garcia et al. 2021).

In the realm of taxonomic classification, both traditional ML and deep learning techniques have showcased promise. Specifically, algorithms such as VirusTaxo, VirFinder, and IDTAXA in the traditional ML category are complemented by others like DeepVirFinder, CHEER, and DeepMicrobes in the deep learning domain (Ren et al. 2017, 2020; Murali et al. 2018; Liang et al. 2020; Shang and Sun 2021; Raju et al. 2022). Each of these methods brings their own strengths and constraints concerning classification precision and recall, but together they collectively furnish a robust instrument for probing into biological diversity and virus taxonomy.

#### Traditional ML-Based Methods

In the domain of taxonomic classification, traditional ML methodologies play a pivotal role, surpassing deep learning techniques in terms of interpretability and computational efficiency, while often matching their performance levels (Alam and Chowdhury 2020). These methods encompass a variety of algorithms, including Naive Bayes, logistic regression, tree-based techniques, and sequence analysis, each offering distinct advantages in addressing taxonomic challenges (Song and Wang 2023).

Specifically, QIIME 2 is renowned for its q2-feature-classifier plugin, which leverages a suite of ML classifiers from scikit-learn to redefine taxonomic classification of marker gene sequences into a structured document classification framework (Bokulich et al. 2018; Bolyen et al. 2019). This plugin’s adaptability allows for sophisticated customization, enabling the integration of diverse feature extraction and transformation methods, alongside the selection of an optimal ML algorithm. At the heart of this plugin is a method that utilizes k-mer counts from reference sequences to train a multinomial Naive Bayes classifier, a technique that notably enhances its capabilities by employing the HashingVectorizer for feature extraction. This advancement extends the k-mer length analysis from the traditional 8-mers used by the RDP Classifier to up to 32-mers, significantly surpassing the conventional classifier's limitations (Wang et al. 2007). Additionally, this method fine-tunes class weights, allowing the classifier to move beyond uniform class prior assumptions and more accurately reflect the natural distribution of taxa in samples, thereby underscoring the balance between interpretability and computational efficiency that traditional ML methods, particularly Naive Bayes, bring to the field of taxonomic classification (Song and Wang 2023).

IDTAXA combines decision tree methods with bootstrap sampling to form an effective tree-based classification system. It builds decision trees to segment data into binary decisions, capturing complex relationships (Murali et al. 2018). Nodes in the tree represent data set features, chosen for their class separation ability, which helps in segmenting the feature space. Bootstrap sampling generates multiple subsets of the original data to create an ensemble of trees, improving accuracy and generalizability. The model is trained by selecting features and splits iteratively and tested on separate data sets, including simulated ones, to evaluate performance. This approach allows IDTAXA to accurately classify complex biological data by leveraging the ensemble of trees. However, tree-based methods may confront challenges such as lengthy training times and recall to parameter selection.

#### Deep Learning–Based Methods

As the demands for complex, detailed, and accurate classification tasks continue to grow within the biological sciences, deep learning, with its convolutional neural networks (CNN), long short-term memory networks (LSTM), and other architectures, has emerged as a crucial component of the solution repertoire (Yang et al. 2022). These methods are adept at automatically extracting features from raw data and capturing complex nonlinearities, significantly enhancing classification accuracy and generalizability compared with traditional ML approaches. Deep learning's application spans genome and protein sequence analysis, as well as specific tasks such as RNA virus classification; despite its computationally intensive training process and the need for meticulous hyperparameter tuning, its strengths in automatic feature learning and complex model construction establish it as a cornerstone in advanced bioinformatics tools.

BERTax is a novel method for DNA sequence classification that relies on deep learning and employs the BERT architecture commonly used in natural language processing (Mock et al. 2022). What sets this method apart is its ability to operate independently of similar sequences in databases, which makes it outstanding for the classification of new species. By segmenting DNA sequences into k-mers as inputs, BERTax trains on these segments to classify sequences across diverse taxonomic levels, ranging from kingdoms to genera. Nonetheless, BERTax may show reduced predictive power at lower taxonomic levels, such as at the genus level.

## Results

### Comparison of Classifier Performance

**Fig. 2.**
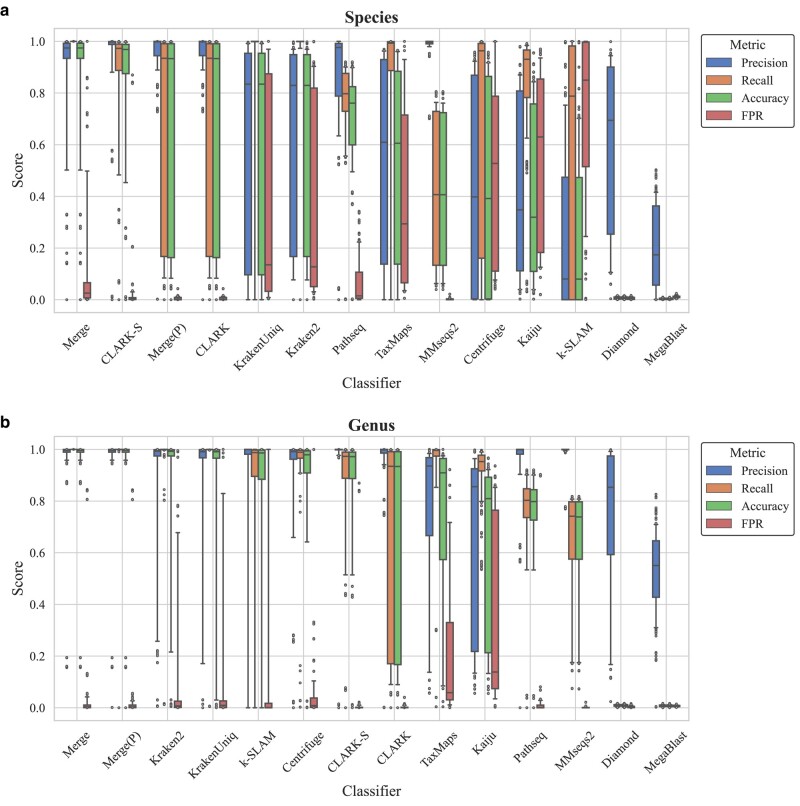
Performance evaluation of DB classification methods. This boxplot illustrates the accuracy, precision, recall, and FPR values for various DB classification approaches on a specific data set. The “Merge” method is referred to as the result integration strategy discussed in this paper, and “Merge(P)” represents our high-precision integration strategy. Accuracy represents the proportion of samples correctly identified by the classifier, precision measures the classifier's ability to correctly label a sample as a specific category, recall is the proportion of all relevant category samples that are identified by the classifier, and the FPR indicates the frequency at which nontarget samples are incorrectly labeled as target categories. The methods are sorted by the median accuracy to facilitate comparison. a) Represents species level and b) represents genus level.

**Fig. 3.**
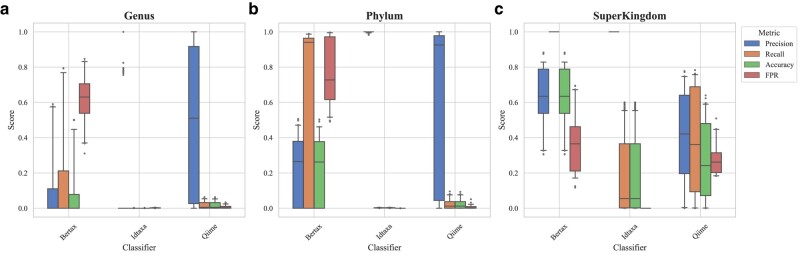
Performance comparison of classification methods based on ML. This figure presents three boxplots, each representing the performance metrics—accuracy, precision, recall, and FPR—across different taxonomic levels: genus, phylum, and superkingdom. a) Represents genus level, b) represents phylum level, and c) represents superkingdom level.

In the current research, we have thoroughly compared the performance of different types of classifiers, aiming to highlight performance disparities between DB classification algorithms and ML classifiers across diverse biological taxonomic levels. Our particular attention was given to the database approaches at the species and genus levels because these taxonomic categories often present challenges and practical relevance in microbial identification (McIntyre et al. 2017; Sczyrba et al. 2017) (refer to Fig. 2). In contrasting ML approaches, we particularly examined their efficacy at the genus, phylum, and superkingdom taxonomic levels (refer to Fig. 3). The selection was made for two primary reasons: Firstly, ML-based classifiers are still evolving, and their effectiveness in fine-grained classification in microbiology, like at the species level, is yet to be improved (Liang et al. 2020; Mock et al. 2022); secondly, given the capability of ML methods in dealing with large and high-dimensional data sets, we anticipate they may excel at higher taxonomic levels like phylum and superkingdom. Such a multitiered comparison will assist us in fully grasping the strengths and weaknesses of the different approaches.

At the species level (refer to Fig. 2a), methods based on databases demonstrate notable disparities in their performance (Sczyrba et al. 2017). Numerous software suites misclassify organisms into incorrect subspecies, severely compromising their accuracy and leading to subpar overall performance. Yet, at the genus level (refer to Fig. 2b), continuously updated methods based on databases have garnered exceptionally good results.

**Fig. 4.**
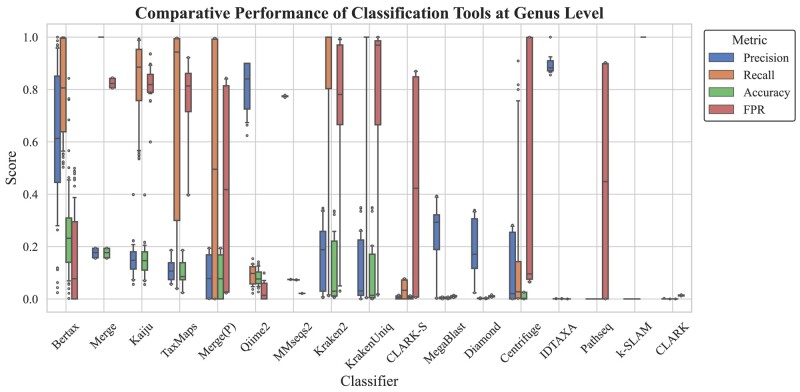
A boxplot comparing the performance of ML and DB methods at the genus level on data not included in training databases, showcasing precision, recall, accuracy, and FPR. The boxplots are sorted by the average accuracy. Details can be found in the legend.

Despite this, at the superkingdom level where they are purportedly strong (see Fig. 3c), ML classification methods still fall short of competing with DB approaches. However, in scenarios where species are absent from databases and not included in the training set, ML classification methods show a significant advantage in accuracy. A box plot (see Fig. 4) now visually compares the classification precision, recall, accuracy, and false positive rate (FPR) of ML and DB methods under these circumstances. Although DB methods demonstrate exceptionally high recall, ML approaches, such as BERTax, generally outperform in precision, accuracy, and FPR. Notably, BERTax ranks first in overall performance metrics. This highlights the significant potential of ML methods in classifying species not represented in databases, showing their capability to effectively handle cases where species are unknown or new to the system. In addition, we conducted experiments with seven different sensitivity settings of DIAMOND, which demonstrated that adjusting sensitivity has a minimal impact on the results (see Supplementary material online).

The Merge method, which consolidates the results from eight database-reliant software tools, stands out for achieving the highest accuracy levels at both species and genus levels in the comprehensive test data set, as shown in Fig. 2. This indicates the method's effectiveness in integrating diverse software outputs to enhance classification precision for species well-represented within databases. However, its precision significantly diminishes when applied to sequences from species not included in the databases, as illustrated in Fig. 4, where it falls below the median precision among the evaluated tools. This downturn in effectiveness can be primarily attributed to the fact that the majority of the individual software tools in the Merge ensemble also misclassify these unknown sequences. Since Merge methodologically selects the consensus or majority classification from its constituent tools, its precision is inherently limited by the accuracy of these individual tools. Consequently, when these tools collectively misclassify an unknown sequence, the Merge method is predisposed to adopt these incorrect classifications, leading to reduced precision. This outcome highlights a fundamental limitation of the Merge approach: its dependency on the accuracy of individual DB tools, which, in the absence of database representation for certain species, often leads to the propagation of classification errors. This scenario underscores the challenge of relying on consensus-based methods for classifying species absent from reference databases, revealing a critical area for improvement in the design and application of integrated classification strategies.

**Fig. 5.**
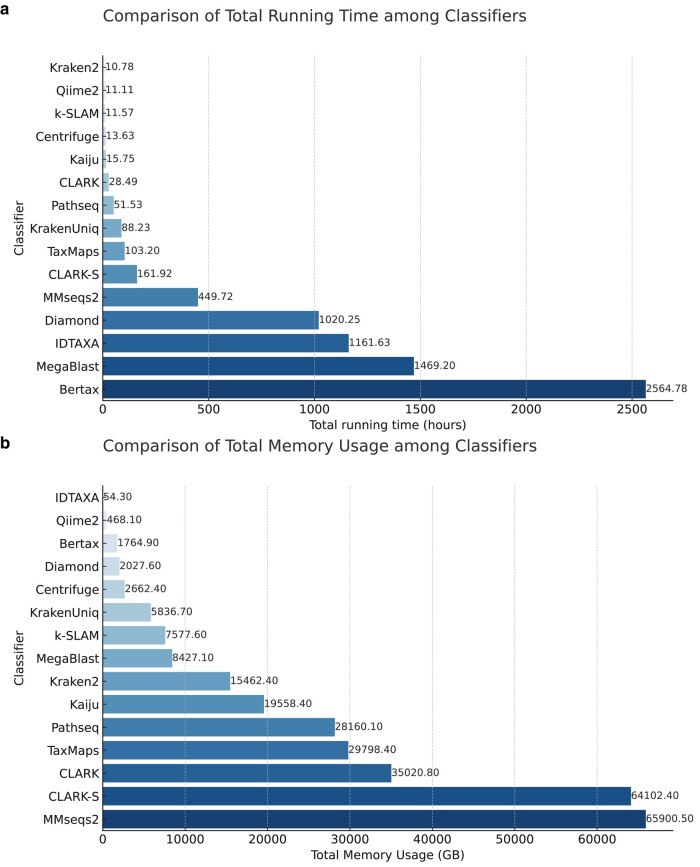
Running resource consumption of classification software. a) Represents the total running time for each software and b) represents the total memory usage for each software.

**Fig. 6.**
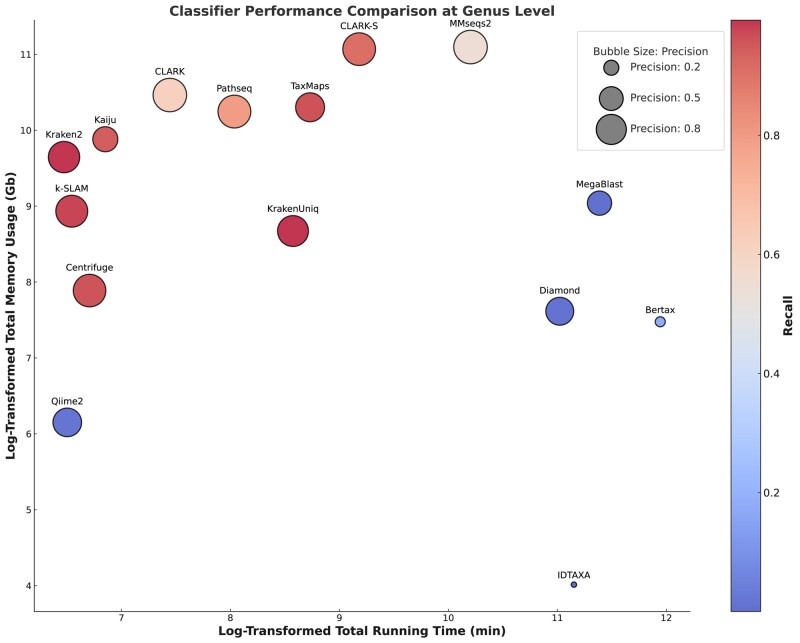
Comprehensive performance comparison of various microbiome classifiers at the genus level. The x-axis and y-axis represent the log-transformed total running time in minutes and total memory usage in gigabytes (Gb), respectively. The size of each bubble indicates the precision of the classifier, while the recall rate is represented by the corresponding values on the scale bar on the right.

Regarding actual runtime and memory usage, a more nuanced understanding can be gained by examining Figs. 5a and 5b and 6. In Fig. 5a, it is evident that ML classification methods often take longer in most cases. Figure 5b further corroborates that these ML methods tend to consume noticeably less memory compared with DB methods. This dichotomy can be attributed to the inherent characteristics of ML algorithms, which often invest more time in model training, thus efficiently conserving computational resources and lowering memory usage during runtime. Consequently, in scenarios where computational resources are constrained but time is not a limiting factor, ML methods could offer significant advantages.

It is noteworthy that while Qiime2 appears to operate with relatively shorter runtimes as depicted in Fig. 5a, the actual time consumed for data processing is significantly longer and is not reflected in the runtime calculations. On the flip side, classification methods based on databases generally show shorter runtimes (Fig. 5a) but consume significantly more memory (Fig. 5b). The primary reason for this is the necessity to load substantial reference databases into memory to facilitate quick searches, thereby significantly increasing memory consumption. In settings where time is of the essence but resources are abundant, these DB methods may be more advantageous.

However, DB classification methods also offer options to reduce memory consumption. For instance, Kraken2 allows for a reduction in memory usage by downsizing the database, with official low-memory versions of the database available, albeit at the expense of a reduced number of identifiable species. KrakenUniq introduces a technique of loading only a small portion of the database into memory at a time for processing, thus diminishing memory requirements. CLARK has developed a lightweight variant, CLARK-l, which reduces memory demands to 4% of the original CLARK requirements by compromising on execution speed and recall. DIAMOND permits the adjustment of block size and the number of seed index chunks processed, enabling a balance between memory usage and performance. MMseqs2 reduces memory demands by segmenting the database.

Despite these solutions offering relief in memory-constrained environments, it is imperative to acknowledge that reducing memory consumption invariably impacts other performance metrics, such as classification speed, accuracy, and the breadth of identifiable species. The selection of the appropriate tool and configuration depends not only on memory limitations but also on the specific requirements and objectives of data processing. Therefore, users must navigate a trade-off among memory usage, execution time, and classification capabilities, opting for the method that best suits their particular scenario.

For a more holistic understanding, Fig. 6 provides an integrated view by plotting the log-transformed runtime against the log-transformed memory usage for each classifier. The size and color of each bubble in Fig. 6 additionally represent the precision and recall metrics, respectively, providing a multifaceted evaluation of classifier performance at the genus level. This comprehensive view aids in better understanding the trade-offs involved and could be instrumental in the informed selection of an appropriate method based on the specific needs of a given study.

### Evaluation of Performance in Results Integration

The strategy of direct integration of classification outcomes, employed in this study, led to a substantial enhancement in the performance of taxonomic classification. By leveraging the strengths of each classification method, we achieved the best results across nearly all evaluation criteria. However, our focus on precision, particularly evident in the Merge(P) strategy, led to exceptionally high precision while slightly reducing recall and accuracy. Notably, the performance of Merge(P) at the species level is very close to that of CLARK, yet it underperforms compared with the Merge strategy at the species level and performs similarly at the genus level.

By using the integration strategy, we not only achieved more comprehensive and precise species identification but also succeeded in significantly minimizing the classification errors that could occur using a single method. Within this strategy, Merge focuses on achieving a balance between precision and recall, resulting in robust overall classification results. In contrast, Merge(P) specifically aims to maximize precision, which leads to exceptionally high precision rates but slightly lowers recall and accuracy. This contrast between the two strategies illustrates the trade-offs involved in optimizing different aspects of taxonomic classification. Despite these trade-offs, neither strategy significantly detracts from the overall performance of the integrated outcomes, demonstrating the efficacy of employing tailored approaches depending on specific classification goals.

It is worth noting that this strategy of direct result integration aims not to optimize or modify individual classification methods but rather relies on the original outcomes of each respective method; this ensures that the integration strategy maintains the unique strengths of each method while simultaneously circumventing any uncertainties that could be introduced through optimization or adjustments.

Furthermore, under this integration strategy, the classification results for some species exhibited such marked improvements that it became evident this could be attributed to the synergistic impact of multiple methods. This synergy allows for the accurate categorization of species that are hard to classify using a single approach.

In summary, this strategy of direct result integration consistently surpassed the performance of any individual method on most evaluation criteria, thereby offering a robust and effective strategy for taxonomic classification in high-throughput sequencing data. This clearly affirms the tremendous potential of employing multiple strategies in parallel and integrating results.

## Discussion

In this research, we have conducted a comprehensive analysis comparing database-driven classification approaches and ML classification approaches. A strategy for integrating various methods to enhance the accuracy of taxonomic classification was also introduced. Through this comparative lens, experimental outcomes reveal that both types of methods have specific advantages and limitations that manifest across different levels of biological taxonomy.

Classification methods relying on databases exhibit notable disparities in performance, particularly at the species and genus levels. This may stem from the incorrect categorization of organisms into the wrong subspecies, consequently lowering the overall accuracy of classification (Martínez-Porchas et al. 2016). However, at the genus level, continuously updated software employing DB methods have yielded exceptionally favorable results. These findings not only validate the efficacy of database-driven methods but also spotlight the challenges they could encounter in species-level classification (Nørskov-Lauritsen 2014).

On the other hand, ML methods show their strengths in specific scenarios, such as when databases are incomplete or when classifying species not included in the training set. Despite these advantages, they generally lag behind in terms of actual classification accuracy when benchmarked against DB methods (Shang and Sun 2021). However, it is important to note that the merits of ML approaches in managing large-scale and high-dimensional data should not be overlooked.

From an operational standpoint, DB methods often exhibit a runtime advantage but require significantly more memory than ML methods. This can be attributed to the necessity for these DB methods to load extensive reference databases into memory for quick retrieval. Conversely, the significant time investment during the training phase for ML methods leads to effective savings in computational resources, thereby reducing memory consumption during actual operation.

Having assessed the strengths and weaknesses of both methods, we devised a new strategy for integrated classification results. The strategy employs a weighted voting mechanism to finalize the classification of each species, thereby effectively exploiting the strengths of each classification method. The experimental findings indicate that this strategy significantly enhances performance in taxonomic classification, leading to more comprehensive and accurate species identification.

It is noteworthy that tools such as FlexTaxD and MetaMeta also aim to enhance classification accuracy by integrating various methods, offering potential value in taxonomic classification. FlexTaxD improves sequence read classification accuracy and precision by modifying and amalgamating both official and custom taxonomic databases (Sundell et al. 2021). This approach is particularly applicable to studies requiring highly customized databases for identifying specific biological communities, thereby addressing complex diversity issues in taxonomy. It enhances the ability to recognize diverse biological information, proving particularly valuable in areas such as bacterial population structure analysis. Meanwhile, MetaMeta provides a comprehensive perspective on species classification by integrating the results of different metagenomic analysis tools (Piro et al. 2017). This is especially effective in handling large-scale metagenomic data, combining the strengths of various analytical tools to offer significant insights into the discovery of new species and understanding the structure and function of microbial communities. However, despite their advantages in improving classification accuracy, both methods support a limited range of classification tools and employ integration techniques different from ours, and they are no longer being updated. Therefore, while these methods have their merits, our proposed integrated approach may be superior in terms of simplicity and effectiveness, although it too has its limitations.

First, the existing merging strategy is somewhat limited, focusing mainly on boosting recall, possibly at the cost of precision. We plan to explore additional merging strategies to enhance the precision of the classification results. Second, while ML methods offer certain benefits in classifying unknown species, our current integrated strategy does not yet incorporate results from ML approaches. As a result, adding outcomes from ML methods to the integration strategy is likely to boost the classification performance for unidentified species. Last, the limited number of species in the data set used for this experiment, along with the less-than-optimal GPU performance, may affect the experiment's reliability and general applicability.

Overall, our study suggests that employing a multistrategy parallel approach and integrating results holds significant potential in the realm of taxonomic classification in high-throughput sequencing data. While each approach has its own set of strengths and weaknesses, we find that by employing an integrated strategy, we can capitalize on the advantages of each to enhance the accuracy of taxonomic classification. In future research, we aim to further refine the integration strategies and improve current classification methods to heighten the accuracy and reliability of species identification.

## Materials and Methods

### Data Set Selection

In this study, we employed the ART Illumina simulator to generate a comprehensive collection of 240 simulated data sets, encompassing a total of 113,282,142 sequences, to assess and compare the efficacy of various species classification methodologies (Huang et al. 2012). ART Illumina was selected for its capability to accurately mimic the sequencing processes of Illumina platforms, such as the HiSeq 2500 and HiSeq 2000, including their characteristic error patterns. The simulated data sets encompassed 30 species validated in the NCBI and SILVA databases, representing a broad spectrum of bacteria and fungi prevalent within microbial communities (Rinke et al. 2021). It is important to note that approximately 10% of the data, corresponding to three species, were intentionally included from outside the databases to simulate real-world scenarios where not all species present in a sample are known or cataloged. The selection of these species reflects their widespread distribution across different ecosystems and the significant variance in their genomic features and ecological traits, laying the groundwork for constructing a challenging microbial community model.

A focal point of our simulation process was the application of read error models, a crucial element in evaluating the accuracy of species classification tools. The error models in ART Illumina, derived from real sequencing data, replicate the specific error rates and types associated with the HiSeq 2500 and HiSeq 2000 platforms, such as base substitutions, insertions, and deletions. These simulated error types account for the randomness and complexity of errors encountered in actual sequencing workflows; for instance, base substitution errors may arise from imperfections in the sequencing instruments’ optical systems, whereas insertions and deletions could result from slippage during the PCR amplification process.

Regarding simulated read lengths and coverage, we established parameters ranging from 100 to 150 base pairs for read lengths and 10 to 30× for coverage, mirroring common settings in actual sequencing experiments based on the performance characteristics of the HiSeq 2500 and HiSeq 2000 platforms. Additionally, to reflect the variability in fragment lengths during library preparation, average fragment lengths were set between 200 and 400, with standard deviations (10, 25, 50) adjusted to simulate this variability. Such parameterization not only reflects the conditions of real sequencing experiments but also enhances the diversity of the simulated data sets, aiding in a comprehensive assessment of the performance of species classification tools under varied conditions.

Through this highly customized simulation process, we were able to generate simulated data sets that are both realistic and embody diverse challenges, providing a solid foundation for evaluating the accuracy, robustness, and applicability of species classification tools in practical scenarios. This approach ensures that the simulated data sets accurately reflect the myriad complexities potentially encountered in real sequencing data, thereby enabling a thorough and effective evaluation of species classification tools.

### Evaluation Methods

In this study, we evaluated the performance of species classifiers using four core metrics: accuracy, precision, recall, and a modified FPR (Saito and Rehmsmeier 2015). Accuracy directly reflects the proportion of species correctly identified by the classifier. Precision focuses on the classifier's ability to correctly label samples as a specific species, while recall measures the proportion of all relevant species samples that the classifier identifies (Ye et al. 2019). Given our simulated data set, where each sequence has a target species, resulting in a true negative count of zero, we redefine FPR as the proportion of sequences incorrectly classified to nontarget taxa relative to the total number of sequences. This adjustment is depicted in the confusion matrix (Table 1), which illustrates the distribution of true positives (TP), false positives (FP), and false negatives (FN) in our classification results. These metrics allow us to comprehensively evaluate the classifier's performance, ensuring its reliability and accuracy in species identification (McIntyre et al. 2017).

**Table 1:** Confusion matrix for DNA sequence classification task using simulated data where each sequence has a target species, resulting in zero true negatives (TN)

	Predicted as target species (PP)	Predicted as nontarget species (PN)
Actual target species (P)	TP: DNA sequence correctly classified as its species	FN: DNA sequence failed to be classified
Actual nontarget species (N)	FP: DNA sequence misclassified as another species	TN: DNA sequence correctly classified as nontarget species

The table categorizes DNA sequences as TP, FP, and FN based on their actual and predicted classifications.

For database-dependent methods, the default operation mode seeks hits that meet the software-defined identity and alignment criteria. Sequences that do not find matches meeting these thresholds are classified as unknown. On the other hand, ML methods classify sequences based on whether their confidence scores are above a certain confidence threshold, again defined by the software. Although some tools allow adjusting this threshold, not every software provides such flexibility. To maintain consistency and fairness in our evaluation, we used the default settings of each classification tool, ensuring a standardized approach to assessing the performance of various methods.

### Applied Taxonomic Classification Methods

For this experiment, we have chosen a range of taxonomic classification methods that are commonly used in practical applications for our assessment. These approaches can be categorized as either DB or ML-based methods, as illustrated in Table 2. To maintain the experiment's fairness and accuracy, all classifiers operated in single-thread mode using default settings, aimed at realizing the best performance for each method, and were run on a single server equipped with a Tesla K80 GPU. Moreover, while all the database methods utilized NCBI's public reference database, we modified it by removing certain species to allow ML methods a chance to showcase their strengths (Nasko et al. 2018).

**Table 2:** Summary of taxonomic classification methods applied in the experiment

Type	Classifier	Synopsis	References
DB	Kraken2	Employs a compact hash table for minimizers to LCA mapping and stores only minimizers, reducing memory needs	(Lu and Salzberg 2020)
	KrakenUniq	Combines k-mer classification with unique k-mer counts using HyperLogLog, supports hierarchical searches and strain/plasmid detection, and integrates extensive viral resources	(Breitwieser et al. 2018)
	Centrifuge	Based on BWT and FM index for genome sequence storage and search, reduces index through compression and redundancy removal, supports variable-length k-mer search	(Kim et al. 2016)
	CLARK	Multilevel short metagenomic read classification; scalable on multicore, low operational requirements; provides confidence scores; supports BAC, transcript, and centromeric region inference	(Ounit et al. 2015)
	CLARK-S	Allows limited mismatches in k-mers to improve recall, introduces discriminative spaced k-mers for precision	(Ounit and Lonardi 2016)
	k-SLAM	Conducts full sequence alignment for gene and variant identification, differing from rapid classifiers that rely solely on k-mers; incorporates pseudo-assembly to enhance accuracy, notably for species with high intra-genus homology	(Ainsworth et al. 2017)
	MegaBLAST	Employs genome database-derived indexing for seed finding, utilizes WindowMasker for soft-masking of repeats, with performance gradually declining in cases of long or highly matched queries	(Morgulis et al. 2008)
	PathSeq	Subtracts human sequences, aligns microbial sequences to detect known/unknown pathogens and resident microorganisms, supports any data set size in parallel computing and cloud environments	(Walker et al. 2018)
	taxMaps	Maps short-read data taxonomically, reduces redundancy and improves query performance with database compression, uses GEM for nonexact match mapping, achieving similar accuracy to BLASTN with reduced time	(Corvelo et al. 2018)
	DIAMOND	Supports ultrafast large-scale protein searches suitable for extensive data sets; utilizes double indexing and multiple spaced seeds for enhanced specificity, optimized by high-performance and cloud computing	(Buchfink et al. 2015)
	Kaiju	Translates metagenomic reads into reading frames for maximum exact matches in a protein database, utilizing BWT for rapid string matching, and employs sparse FM index and adjustable suffix arrays to minimize memory usage	(Menzel et al. 2016)
	MMseqs2	Parallelized for iterative profile-to-sequence and sequence-to-profile searches, capable of handling large data sets, offers clustering to reduce database redundancy, saving storage and computational resources	(Steinegger and Söding 2017)
ML	BERTax	Uses deep neural network and natural language processing to classify DNA sequences’ superkingdom and phylum without database relatives; excels with novel organisms and can classify any genome region	(Mock et al. 2022)
	IDTAXA	Uses ML to reduce overclassification and enhance accuracy, fewer overclassifications with missing data, suitable for varied sequence lengths, autocorrects errors in reference taxonomies, part of R’s DECIPHER package	(Murali et al. 2018)
	QIIME 2	Integrates various feature classifiers and databases for species annotation, supports complex statistical analyses for biodiversity exploration, offers data visualization tools for result presentation, and has a plugin architecture for easy extension of new features	(Bolyen et al. 2019)

### Result Integration

To enhance the reliability and accuracy of species classification, we adopted an integration strategy that consolidates the results from multiple classification methods. The eight selected methods—Kraken2, KrakenUniq, Centrifuge, CLARK, CLARK-S, k-SLAM, MegaBLAST, and Kaiju—were chosen for their rapid processing capabilities and their proficiency in generating definitive classification results for individual sequences. Despite their varied focal points, a common feature among these methods is their reliance on sequence-level precision over phylogenetic tree structures or direct abundance outputs.

**Fig. 7.**
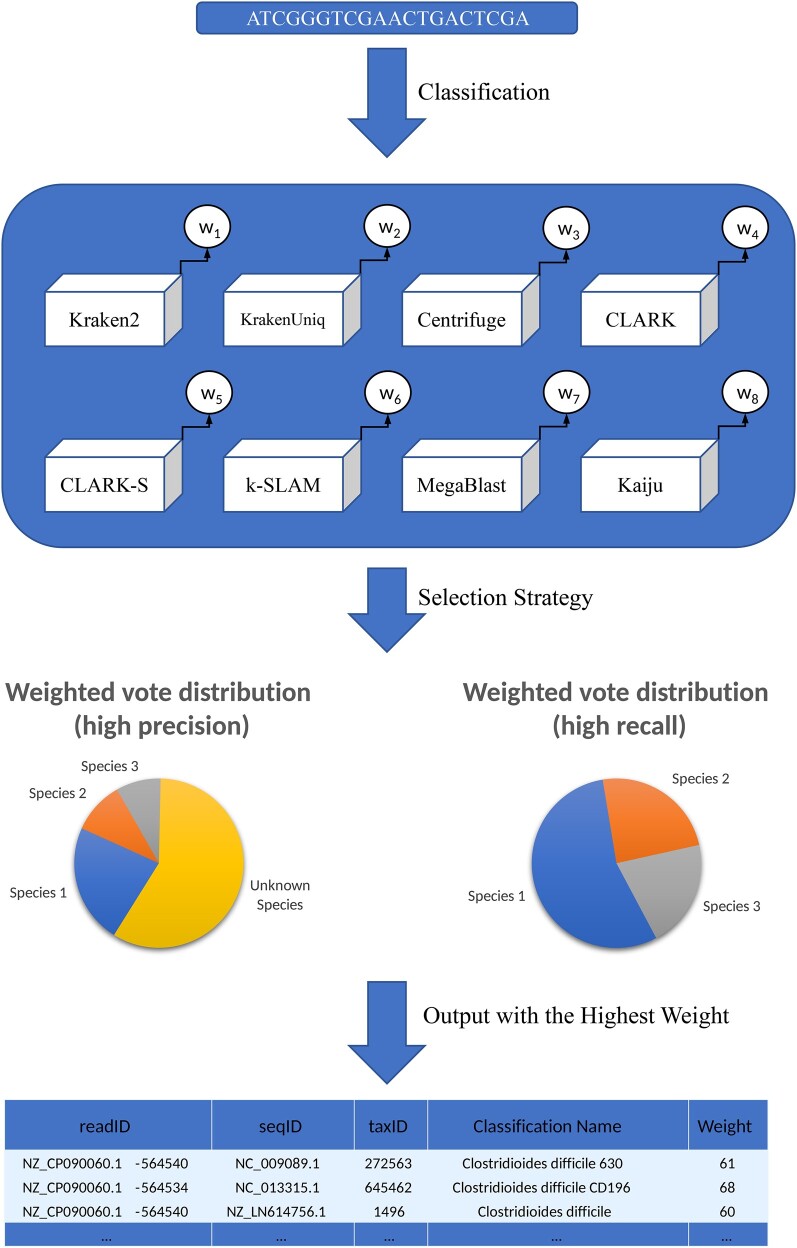
The process of integration using the weighted voting method. The figure shows how the final classification outcome is chosen from among various classification methods using weighted voting. Each classification method's weight (W) is founded on performance indicators like accuracy, recall rate, and the like. The classification outcomes with high recall and precision are represented using two pie charts. The final classification outcomes are then tabulated below the figure.

In the process of integrating these classification outcomes, we devised a weighted voting system (as illustrated in Fig. 7), where each software's result for every sequence (typically in the form of a taxonomic identifier, or taxid) is voted on based on assigned weights, with the highest weighted outcome being selected. The allocation of weights was based on tests conducted on a separate simulated data set, distinct from the one used in the current experiment. Although this weight distribution underwent preliminary testing and optimization, we acknowledge that it does not represent an optimal solution but rather serves as an exploratory attempt. Hence, in our description, we specifically emphasize that the weight assignment was based on “preliminary settings derived from testing on an alternate simulated data set, indicating a provisional and exploratory nature.”

Furthermore, we encourage users to customize weights according to the specific characteristics of their data and research objectives. This flexibility is crucial for accommodating diverse research contexts and aims.

Our integration strategy explores two distinct paths: one leaning toward high recall, by excluding results that were not successfully classified and retaining only those unanimously agreed upon by the methods, and another leaning toward high precision, which considers all results, including unsuccessful classifications. Notably, when the aggregated weight of the “unknown” category is high, this approach classifies the corresponding sequence as unknown. By embracing the diversity of these strategies and providing researchers with the ability to customize weights, we aim to optimize the overall effectiveness of species classification, leveraging the strengths of each method to their fullest.

## Supplementary Material

evae102_Supplementary_Data

## Data Availability

All data supporting the findings of this study are available within the article and its supplementary materials, Supplementary Material online. The data sets generated and analyzed during the current study are available in the Zenodo repository at https://doi.org/10.5281/zenodo.10666087. The data analysis scripts and code used in this study are available in the GitHub repository at https://github.com/LoadStar822/Genome-Classifier-Performance-Evaluator.

## References

[evae102-B1] Ainsworth D, Sternberg MJE, Raczy C, Butcher SA. k-SLAM: accurate and ultra-fast taxonomic classification and gene identification for large metagenomic data sets. Nucleic Acids Res. 2017:45(4):1649–1656. 10.1093/nar/gkw1248. 27965413 PMC5389551

[evae102-B2] Alam MNU, Chowdhury UF. Short k-mer abundance profiles yield robust machine learning features and accurate classifiers for RNA viruses. PLoS One 2020:15(9):e0239381. 10.1371/journal.pone.0239381. 32946529 PMC7500682

[evae102-B3] Ames SK, Hysom DA, Gardner SN, Lloyd GS, Gokhale MB, Allen JE. Scalable metagenomic taxonomy classification using a reference genome database. Bioinforma. 2013:29(18):2253–2260. 10.1093/bioinformatics/btt389. PMC3753567 23828782

[evae102-B4] Bartlett P, Eberhardt U, Schütz N, Beker HJ. Species determination using AI machine-learning algorithms: *Hebeloma* as a case study. IMA Fungus. 2022:13(1):13. 10.1186/s43008-022-00099-x. 35773719 PMC9245212

[evae102-B5] Blanco-Míguez A, Beghini F, Cumbo F, McIver LJ, Thompson KN, Zolfo M, Manghi P, Dubois L, Huang KD, Thomas AM, et al Extending and improving metagenomic taxonomic profiling with uncharacterized species using MetaPhlAn 4. Nat Biotechnol. 2023:41(11):1633–1644. 10.1038/s41587-023-01688-w. 36823356 PMC10635831

[evae102-B6] Bokulich NA, Kaehler BD, Rideout JR, Dillon M, Bolyen E, Knight R, Huttley GA, Gregory Caporaso J. Optimizing taxonomic classification of marker-gene amplicon sequences with QIIME 2's q2-feature-classifier plugin. Microbiome 2018:6(1):90. 10.1186/s40168-018-0470-z. 29773078 PMC5956843

[evae102-B7] Bolyen E, Rideout JR, Dillon MR, Bokulich NA, Abnet CC, Al-Ghalith GA, Alexander H, Alm EJ, Arumugam M, Asnicar F, et al Reproducible, interactive, scalable and extensible microbiome data science using QIIME 2. Nat Biotechnol. 2019:37(8):852–857. 10.1038/s41587-019-0209-9. 31341288 PMC7015180

[evae102-B8] Bonin N, Doster E, Worley H, Pinnell LJ, Bravo JE, Ferm P, Marini S, Prosperi M, Noyes N, Morley PS, et al MEGARes and AMR++, v3.0: an updated comprehensive database of antimicrobial resistance determinants and an improved software pipeline for classification using high-throughput sequencing. Nucleic Acids Res. 2023:51(D1):D744–D752. 10.1093/nar/gkac1047. 36382407 PMC9825433

[evae102-B9] Borba VH, Martin C, Machado-Silva JR, Xavier SCC, de Mello FL, Iñiguez AM. Machine learning approach to support taxonomic species discrimination based on helminth collections data. Parasit Vectors. 2021:14(1):230. 10.1186/s13071-021-04721-6. 33933139 PMC8088700

[evae102-B10] Breitwieser FP, Baker DN, Salzberg SL. KrakenUniq: confident and fast metagenomics classification using unique k-mer counts. Genome Biol. 2018:19(1):1–10. 10.1186/s13059-018-1568-0. 30445993 PMC6238331

[evae102-B11] Buchfink B, Xie C, Huson DH. Fast and sensitive protein alignment using DIAMOND. Nat Methods. 2015:12(1):59–60. 10.1038/nmeth.3176. 25402007

[evae102-B12] Corvelo A, Clarke WE, Robine N, Zody MC. taxMaps: comprehensive and highly accurate taxonomic classification of short-read data in reasonable time. Genome Res. 2018:28(5):751–758. 10.1101/gr.225276.117. 29588360 PMC5932614

[evae102-B13] Dubinkina VB, Ischenko DS, Ulyantsev VI, Tyakht AV, Alexeev DG. Assessment of k-mer spectrum applicability for metagenomic dissimilarity analysis. BMC Bioinformatics. 2016:17(1):38. 10.1186/s12859-015-0875-7. 26774270 PMC4715287

[evae102-B14] Eisenhofer R, Weyrich LS. Assessing alignment-based taxonomic classification of ancient microbial DNA. PeerJ. 2019:7:e6594. 10.7717/peerj.6594. 30886779 PMC6420809

[evae102-B15] Furstenau TN, Schneider T, Shaffer I, Vazquez AJ, Sahl J, Fofanov V. MTSv: rapid alignment-based taxonomic classification and high-confidence metagenomic analysis. PeerJ. 2022:10:e14292. 10.7717/peerj.14292. 36389404 PMC9651046

[evae102-B16] Gao X, Lin H, Revanna K, Dong Q. A Bayesian taxonomic classification method for 16S rRNA gene sequences with improved species-level accuracy. BMC Bioinformatics. 2017:18(1):247. 10.1186/s12859-017-1670-4. 28486927 PMC5424349

[evae102-B17] Garcia BJ, Simha R, Garvin M, Furches A, Jones P, Gazolla JGFM, Hyatt PD, Schadt CW, Pelletier D, Jacobson D. A k-mer based approach for classifying viruses without taxonomy identifies viral associations in human autism and plant microbiomes. Comput Struct Biotechnol J. 2021:19:5911–5919. 10.1016/j.csbj.2021.10.029. 34849195 PMC8605058

[evae102-B18] Gardiner L-J, Haiminen N, Utro F, Parida L, Seabolt E, Krishna R, Kaufman JH. Re-purposing software for functional characterization of the microbiome. Microbiome 2021:9(1):4. 10.1186/s40168-020-00971-1. 33422152 PMC7797099

[evae102-B19] Han G-B, Cho D-H. Genome classification improvements based on k-mer intervals in sequences. Genomics 2019:111(6):1574–1582. 10.1016/j.ygeno.2018.11.001. 30439480

[evae102-B20] Hassemer G, Bruun-Lund S, Shipunov AB, Briggs BG, Meudt HM, Rønsted N. The application of high-throughput sequencing for taxonomy: the case of *Plantago* subg. *Plantago* (Plantaginaceae). Mol Phylogenet Evol. 2019:138:156–173. 10.1016/j.ympev.2019.05.013. 31112781

[evae102-B21] Huang W, Li L, Myers JR, Marth GT. ART: a next-generation sequencing read simulator. Bioinformatics. 2012:28(4):593–594. 10.1093/bioinformatics/btr708. 22199392 PMC3278762

[evae102-B22] Hugenholtz P, Chuvochina M, Oren A, Parks DH, Soo RM. Prokaryotic taxonomy and nomenclature in the age of big sequence data. ISME J. 2021:15(7):1879–1892. 10.1038/s41396-021-00941-x. 33824426 PMC8245423

[evae102-B23] Johnson JS, Spakowicz DJ, Hong B-Y, Petersen LM, Demkowicz P, Chen L, Leopold SR, Hanson BM, Agresta HO, Gerstein M, et al Evaluation of 16S rRNA gene sequencing for species and strain-level microbiome analysis. Nat Commun. 2019:10(1):5029. 10.1038/s41467-019-13036-1. 31695033 PMC6834636

[evae102-B24] Kim D, Song L, Breitwieser FP, Salzberg SL. Centrifuge: rapid and sensitive classification of metagenomic sequences. Genome Res. 2016:26(12):1721–1729. 10.1101/gr.210641.116. 27852649 PMC5131823

[evae102-B25] Kim HY, Seo J, Kim T-H, Shim B, Cha SM, Yu S. Pyrosequencing-based assessment of microbial community shifts in leachate from animal carcass burial lysimeter. Sci Total Environ. 2017:587–588:232–239. 10.1016/j.scitotenv.2017.02.126. 28249748

[evae102-B26] Lan Y, Rosen G, Hershberg R. Marker genes that are less conserved in their sequences are useful for predicting genome-wide similarity levels between closely related prokaryotic strains. Microbiome 2016:4(1):18. 10.1186/s40168-016-0162-5. 27138046 PMC4853863

[evae102-B27] Liang Q, Bible PW, Liu Y, Zou B, Wei L. DeepMicrobes: taxonomic classification for metagenomics with deep learning. NAR Genomics Bioinforma. 2020:2(1):lqaa009. 10.1093/nargab/lqaa009. PMC7671387 33575556

[evae102-B28] Liu X, Yu Y, Liu J, Elliott CF, Qian C, Liu J. A novel data structure to support ultra-fast taxonomic classification of metagenomic sequences with k-mer signatures. Bioinformatics. 2018:34(1):171–178. 10.1093/bioinformatics/btx432. 29036588 PMC5870563

[evae102-B29] Lu J, Salzberg SL. Ultrafast and accurate 16S rRNA microbial community analysis using Kraken 2. Microbiome 2020:8(1):124. 10.1186/s40168-020-00900-2. 32859275 PMC7455996

[evae102-B30] Martínez-Porchas M, Villalpando-Canchola E, Vargas-Albores F. Significant loss of sensitivity and specificity in the taxonomic classification occurs when short 16S rRNA gene sequences are used. Heliyon. 2016:2(9):e00170. 10.1016/j.heliyon.2016.e00170. 27699286 PMC5037269

[evae102-B31] McIntyre ABR, Ounit R, Afshinnekoo E, Prill RJ, Hénaff E, Alexander N, Minot SS, Danko D, Foox J, Ahsanuddin S, et al Comprehensive benchmarking and ensemble approaches for metagenomic classifiers. Genome Biol. 2017:18(1):182. 10.1186/s13059-017-1299-7. 28934964 PMC5609029

[evae102-B32] Menzel P, Ng KL, Krogh A. Fast and sensitive taxonomic classification for metagenomics with Kaiju. Nat Commun. 2016:7(1):11257. 10.1038/ncomms11257. 27071849 PMC4833860

[evae102-B33] Mock F, Kretschmer F, Kriese A, Böcker S, Marz M. Taxonomic classification of DNA sequences beyond sequence similarity using deep neural networks. Proc Natl Acad Sci U S A. 2022:119(35):e2122636119. 10.1073/pnas.2122636119. PMC9436379 36018838

[evae102-B34] Morgulis A, Coulouris G, Raytselis Y, Madden TL, Agarwala R, Schäffer AA. Database indexing for production MegaBLAST searches. Bioinformatics. 2008:24(16):1757–1764. 10.1093/bioinformatics/btn322. 18567917 PMC2696921

[evae102-B35] Murali A, Bhargava A, Wright ES. IDTAXA: a novel approach for accurate taxonomic classification of microbiome sequences. Microbiome 2018:6(1):140. 10.1186/s40168-018-0521-5. 30092815 PMC6085705

[evae102-B36] Nasko DJ, Koren S, Phillippy AM, Treangen TJ. RefSeq database growth influences the accuracy of k-mer-based lowest common ancestor species identification. Genome Biol. 2018:19(1):165. 10.1186/s13059-018-1554-6. 30373669 PMC6206640

[evae102-B37] Nooij S, Schmitz D, Vennema H, Kroneman A, Koopmans MPG. Overview of virus metagenomic classification methods and their biological applications. Front Microbiol. 2018:9:749. 10.3389/fmicb.2018.00749. 29740407 PMC5924777

[evae102-B38] Nørskov-Lauritsen N . Classification, identification, and clinical significance of *Haemophilus* and *Aggregatibacter* species with host specificity for humans. Clin Microbiol Rev. 2014:27(2):214–240. 10.1128/CMR.00103-13. 24696434 PMC3993099

[evae102-B39] Ounit R, Lonardi S. Higher classification sensitivity of short metagenomic reads with CLARK-S. Bioinformatics. 2016:32(24):3823–3825. 10.1093/bioinformatics/btw542. 27540266

[evae102-B40] Ounit R, Wanamaker S, Close TJ, Lonardi S. CLARK: fast and accurate classification of metagenomic and genomic sequences using discriminative k-mers. BMC Genomics. 2015:16(1):236. 10.1186/s12864-015-1419-2. 25879410 PMC4428112

[evae102-B41] Parks DH, Chuvochina M, Chaumeil P-A, Rinke C, Mussig AJ, Hugenholtz P. A complete domain-to-species taxonomy for Bacteria and Archaea. Nat Biotechnol. 2020:38(9):1079–1086. 10.1038/s41587-020-0501-8. 32341564

[evae102-B42] Piro VC, Dadi TH, Seiler E, Reinert K, Renard BY. Ganon: precise metagenomics classification against large and up-to-date sets of reference sequences. Bioinformatics. 2020:36(Supplement_1):i12–i20. 10.1093/bioinformatics/btaa458. 32657362 PMC7355301

[evae102-B43] Piro VC, Matschkowski M, Renard BY. MetaMeta: integrating metagenome analysis tools to improve taxonomic profiling. Microbiome 2017:5(1):101. 10.1186/s40168-017-0318-y. 28807044 PMC5557516

[evae102-B44] Portik DM, Brown CT, Pierce-Ward NT. Evaluation of taxonomic classification and profiling methods for long-read shotgun metagenomic sequencing datasets. BMC Bioinformatics. 2022:23(1):541. 10.1186/s12859-022-05103-0. 36513983 PMC9749362

[evae102-B45] Raju RS, Al Nahid A, Chondrow Dev P, Islam R. VirusTaxo: taxonomic classification of viruses from the genome sequence using k-mer enrichment. Genomics 2022:114(4):110414. 10.1016/j.ygeno.2022.110414. 35718090

[evae102-B46] Ren J, Ahlgren NA, Lu YY, Fuhrman JA, Sun F. VirFinder: a novel k-mer based tool for identifying viral sequences from assembled metagenomic data. Microbiome 2017:5(1):69. 10.1186/s40168-017-0283-5. 28683828 PMC5501583

[evae102-B47] Ren J, Song K, Deng C, Ahlgren NA, Fuhrman JA, Li Y, Xie X, Poplin R, Sun F, et al Identifying viruses from metagenomic data using deep learning. Quant Biol. 2020:8(1):64–77. 10.1007/s40484-019-0187-4. 34084563 PMC8172088

[evae102-B48] Rinke C, Chuvochina M, Mussig AJ, Chaumeil P-A, Davín AA, Waite DW, Whitman WB, Parks DH, Hugenholtz P. A standardized archaeal taxonomy for the Genome Taxonomy Database. Nat Microbiol. 2021:6(7):946–959. 10.1038/s41564-021-00918-8. 34155373

[evae102-B49] Saito T, Rehmsmeier M. The precision-recall plot is more informative than the ROC plot when evaluating binary classifiers on imbalanced datasets. PLoS One 2015:10(3):e0118432. 10.1371/journal.pone.0118432. 25738806 PMC4349800

[evae102-B50] Sczyrba A, Hofmann P, Belmann P, Koslicki D, Janssen S, Dröge J, Gregor I, Majda S, Fiedler J, Dahms E, et al Critical assessment of metagenome interpretation—a benchmark of computational metagenomics software. Nat Methods. 2017:14(11):1063–1071. 10.1038/nmeth.4458. 28967888 PMC5903868

[evae102-B51] Shang J, Sun Y. CHEER: HierarCHical taxonomic classification for viral mEtagEnomic data via deep leaRning. Methods 2021:189:95–103. 10.1016/j.ymeth.2020.05.018. 32454212 PMC7255349

[evae102-B52] Shaw J, Yu YW. Theory of local k-mer selection with applications to long-read alignment. Bioinforma. 2022:38(20):4659–4669. 10.1093/bioinformatics/btab790. PMC9563685 36124869

[evae102-B53] Song G, Wang Q. Species classification from hyperspectral leaf information using machine learning approaches. Ecol Inform. 2023:76:102141. 10.1016/j.ecoinf.2023.102141.

[evae102-B54] Steinegger M, Söding J. MMseqs2 enables sensitive protein sequence searching for the analysis of massive data sets. Nat Biotechnol. 2017:35(11):1026–1028. 10.1038/nbt.3988. 29035372

[evae102-B55] Sundell D, Öhrman C, Svensson D, Karlsson E, Brindefalk B, Myrtennäs K, Ahlinder J, Antwerpen MH, Walter MC, Forsman M, et al FlexTaxD: flexible modification of taxonomy databases for improved sequence classification. Bioinformatics. 2021:37(21):3932–3933. 10.1093/bioinformatics/btab621. 34469515

[evae102-B56] Tovo A, Menzel P, Krogh A, Cosentino Lagomarsino M, Suweis S. Taxonomic classification method for metagenomics based on core protein families with Core-Kaiju. Nucleic Acids Res. 2020:48(16):e93. 10.1093/nar/gkaa568. 32633756 PMC7498351

[evae102-B57] Uyaguari-Diaz MI, Chan M, Chaban BL, Croxen MA, Finke JF, Hill JE, Peabody MA, Van Rossum T, Suttle CA, Brinkman FSL, et al A comprehensive method for amplicon-based and metagenomic characterization of viruses, bacteria, and eukaryotes in freshwater samples. Microbiome 2016:4(1):20. 10.1186/s40168-016-0166-1. 27391119 PMC5011856

[evae102-B58] Van Etten J, Stephens TG, Bhattacharya D. A k-mer-based approach for phylogenetic classification of taxa in environmental genomic data. Syst Biol. 2023:72(5):1101–1118. 10.1093/sysbio/syad037. 37314057

[evae102-B59] Vicente Dos Santos V, Tixier M-S. Which molecular markers for assessing which taxonomic level? The case study of the mite family Phytoseiidae (Acari: Mesostigmata). Cladistics. 2017:33(3):251–267. 10.1111/cla.12166. 34715727

[evae102-B60] Vinje H, Liland KH, Almøy T, Snipen L. Comparing K-mer based methods for improved classification of 16S sequences. BMC Bioinformatics. 2015:16(1):205. 10.1186/s12859-015-0647-4. 26130333 PMC4487979

[evae102-B61] Walker MA, Pedamallu CS, Ojesina AI, Bullman S, Sharpe T, Whelan CW, Meyerson M. GATK PathSeq: a customizable computational tool for the discovery and identification of microbial sequences in libraries from eukaryotic hosts. Bioinformatics. 2018:34(24):4287–4289. 10.1093/bioinformatics/bty501. 29982281 PMC6289130

[evae102-B62] Wang Q, Garrity GM, Tiedje JM, Cole JR. Naïve Bayesian classifier for rapid assignment of rRNA sequences into the new bacterial taxonomy. Appl Environ Microbiol. 2007:73(16):5261–5267. 10.1128/AEM.00062-07. 17586664 PMC1950982

[evae102-B63] Wood DE, Salzberg SL. Kraken: ultrafast metagenomic sequence classification using exact alignments. Genome Biol. 2014:15(3):R46. 10.1186/gb-2014-15-3-r46. 24580807 PMC4053813

[evae102-B64] Wright RJ, Comeau AM, Langille MGI. From defaults to databases: parameter and database choice dramatically impact the performance of metagenomic taxonomic classification tools. Microb Genomics. 2023:9(3):mgen000949. 10.1099/mgen.0.000949. PMC10132073 36867161

[evae102-B65] Yang C, Zheng Y, Tan S, Meng G, Rao W, Yang C, Bourne DG, O’Brien PA, Xu J, Liao S, et al Efficient COI barcoding using high throughput single-end 400 bp sequencing. BMC Genomics. 2020:21(1):862. 10.1186/s12864-020-07255-w. 33276723 PMC7716423

[evae102-B66] Yang C-H, Wu K-C, Chuang L-Y, Chang H-W. DeepBarcoding: deep learning for species classification using DNA barcoding. IEEE/ACM Trans Comput Biol Bioinform. 2022:19(4):2158–2165. 10.1109/TCBB.2021.3056570. 33600318

[evae102-B67] Ye SH, Siddle KJ, Park DJ, Sabeti PC. Benchmarking metagenomics tools for taxonomic classification. Cell 2019:178(4):779–794. 10.1016/j.cell.2019.07.010. 31398336 PMC6716367

[evae102-B68] Zhang P, Liu H, Wei Y, Zhai Y, Tian Q, Zou Q. FMAlign2: a novel fast multiple nucleotide sequence alignment method for ultralong datasets. Bioinformatics. 2024:40(1):btae014. 10.1093/bioinformatics/btae014. 38200554 PMC10809904

