# Supplementary figures and images for: Application and Comparison of Machine Learning and Database-Based Methods in Taxonomic Classification of High-Throughput Sequencing Data
Source: Genome Biol Evol. 2024 May 15;16(5):evae102. doi: 10.1093/gbe/evae102 (PMC11135637; doi:10.1093/gbe/evae102)

Compare the precision and recall of the seven sensitivities of diamond at the genus level

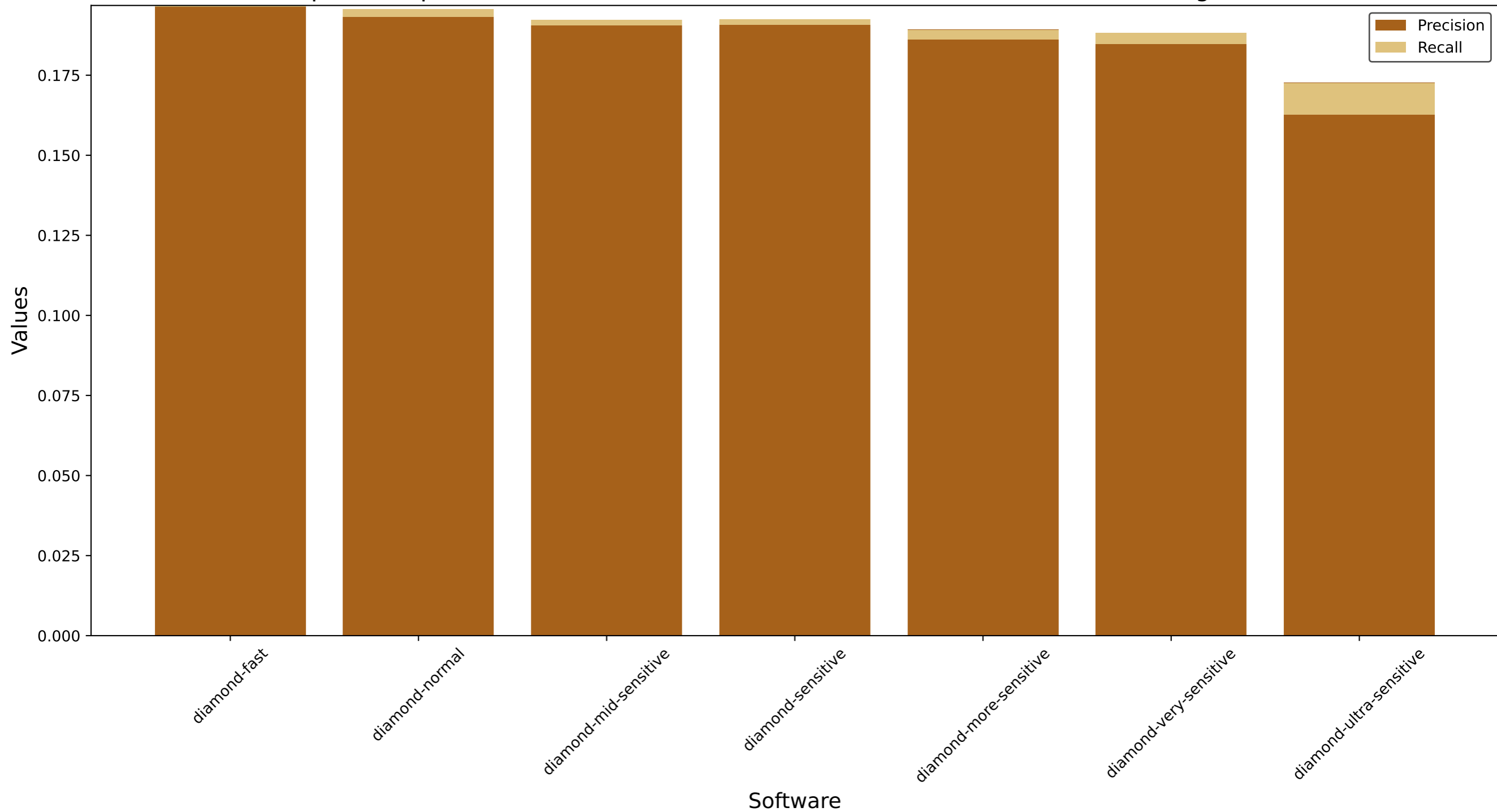

Supplement: evae102_Supplementary_Data [file evae102_supplementary_data.pdf]
